# ClockstaRX: Testing Molecular Clock Hypotheses With Genomic Data

**DOI:** 10.1093/gbe/evae064

**Published:** 2024-03-25

**Authors:** David A Duchêne, Sebastián Duchêne, Josefin Stiller, Rasmus Heller, Simon Y W Ho

**Affiliations:** Center for Evolutionary Hologenomics, University of Copenhagen, Copenhagen 1352, Denmark; Section of Epidemiology, Department of Public Health, University of Copenhagen, Copenhagen 1352, Denmark; Department of Microbiology and Immunology, Peter Doherty Institute for Infection and Immunity, University of Melbourne, Melbourne, VIC 3010, Australia; Villum Centre for Biodiversity Genomics, University of Copenhagen, 2100 Copenhagen, Denmark; Section for Computational and RNA Biology, Department of Biology, University of Copenhagen, Copenhagen 2100, Denmark; School of Life and Environmental Sciences, University of Sydney, Sydney, NSW 2006, Australia

**Keywords:** evolutionary rate, molecular clock, rate heterogeneity, phylogenomics, ClockstaR

## Abstract

Phylogenomic data provide valuable opportunities for studying evolutionary rates and timescales. These analyses require theoretical and statistical tools based on molecular clocks. We present ClockstaRX, a flexible platform for exploring and testing evolutionary rate signals in phylogenomic data. Here, information about evolutionary rates in branches across gene trees is placed in Euclidean space, allowing data transformation, visualization, and hypothesis testing. ClockstaRX implements formal tests for identifying groups of loci and branches that make a large contribution to patterns of rate variation. This information can then be used to test for drivers of genomic evolutionary rates or to inform models for molecular dating. Drawing on the results of a simulation study, we recommend forms of data exploration and filtering that might be useful prior to molecular-clock analyses.

**Significance**

Evolutionary rates are routinely inferred in phylogenomic dating and in studies of the fundamental drivers of molecular evolution. However, resolving the patterns of evolutionary rate variation across large numbers of taxa and whole genomes is not straightforward. These patterns can be identified using methods based on molecular clock theory, which provides specific expectations of variation in rates across branches and genes. With a major software upgrade, we introduce ClockstaRX, a tool for comprehensive hypothesis testing and visualization of evolutionary rates in phylogenomic data.

The molecular clock provides the foundation for studies of evolutionary rates and timescales. In phylogenetic analyses of divergence times, evolutionary rate variation across genes and lineages (i.e. phylogenetic tree branches) is often taken into account using molecular clock models (Ho and Duchêne 2014; dos Reis et al. 2016). In addition, the causes of variation in evolutionary rates have been studied widely, and include the environment (Gillman et al. 2009), life history (Bromham 2009; Iglesias-Carrasco et al. 2019), and selection across the genome (Drummond and Wilke 2008, Yang and Gaut 2011). Phylogenomics offers increasing opportunities to study evolutionary timescales and rates (Ho 2014), but the large size and complexity of these data sets call for dedicated tools for visualization and model comparison.

To study evolutionary rate variation, it is convenient to partition the biological influences on rates into lineage effects, gene effects, and gene-by-lineage interactions (Gillespie 1991; Gaut et al. 2011; Gillespie 1989). Lineage effects are those that drive changes in evolutionary rates across the whole genome in a given lineage (Bromham 2011), such as differences in generation time (e.g. Hua et al. 2015) or metabolic rate (e.g. Montoya et al. 2022). Gene effects are those that lead to variation in rates across loci, as in the case of differing selective constraints among coding and noncoding DNA (e.g. Hughes and Yeager 1997; Laroche et al. 1997). Lastly, gene-by-lineage interactions occur when rates vary in subsets of genes in subsets of lineages (Takahata 1987; Cutler 2000; Bedford and Hartl 2008). For instance, a subset of loci might experience a new selective constraint and consequent reduction in evolutionary rate in a subset of the phylogenetic tree branches being studied.

In genome-scale data, disentangling evolutionary rate variation has various challenges, including accounting for gene-tree discordance (Mendes and Hahn 2016), in describing high-dimensional phylogenetic data (Duchêne et al. 2018a; Duchêne et al. 2018b; Smith 2022), and when formulating meaningful biological hypotheses (Smith and Eyre-Walker 2003). One approach to the statistical analysis of rate variation is through comparison of “pacemaker” models of genomic evolution (Wolf et al. 2009; Snir et al. 2012). These models extend the “universal molecular clock” in which a single evolutionary rate dominates all genes and all phylogenetic tree branches.

In the “universal pacemaker” model, locus rates can be described as a single variable or dimension that represents their relative rates (Fig. 1a). Meanwhile, the “degenerate multiple pacemaker” model asserts that all loci have mutually independent patterns of among-lineage rate variation, such that their branch rates are fully uncorrelated (Snir et al. 2012). These simple models of rate variation are readily usable for phylogenetic inference and are known as “branch linkage models”. Empirical data frequently support a universal pacemaker (Duchêne et al. 2020), suggesting that there are strong correlations in rates across loci.

**Fig. 1.**
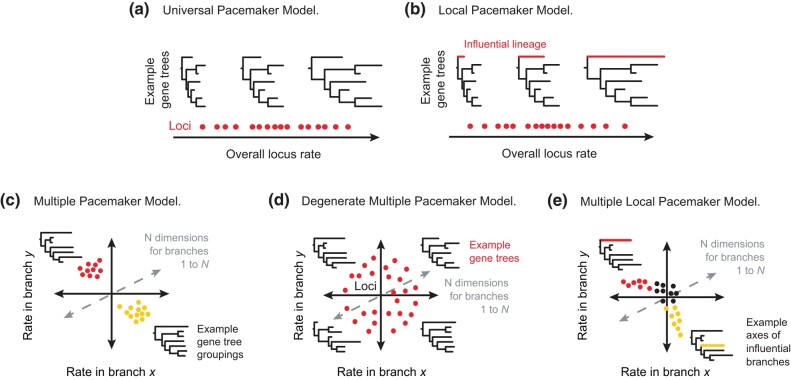
Hypotheses that can be evaluated in a Euclidean space of evolutionary rates with *N* dimensions for branches in a species tree. (a) Universal pacemaker model. Loci have evolutionary rates that covary across branches, such that all variation can be explained by a single summary dimension that minimizes the variance across Euclidean space. (b) Local pacemaker model. A subset of branches has a disproportionate influence on the overall variation in rates, such that they have high variance across the summarized space. (c) Multiple pacemaker model. Loci share patterns of among-lineage rate heterogeneity that are distinct from those of other groups of loci. All loci within a cluster are affected equally such that they group across space. (d) Degenerate multiple pacemaker model. Each locus has its own pattern of among-lineage rate heterogeneity, such that the variables are completely uncorrelated. This model can serve as a null hypothesis. (e) Multiple local pacemaker model. Loci vary in their contribution to the variance in subsets of branches, such that they follow a continuous trend across space.

Here we present ClockstaRX, a comprehensive tool for molecular clock visualization and hypothesis testing for genome-scale data sets. This software provides a wide-ranging advance from its predecessor, ClockstaR (Duchêne et al. 2014). We define the “multiple pacemaker” model as a cornerstone of the predecessor software, which assumed that loci can be grouped into “clusters” of rate patterns where all loci within a cluster are affected equally by a set of drivers of rate variation (Fig. 1c).

In ClockstaRX, we extend this model to test for “multiple local pacemaker” models, where a subset of branches can have various degrees of heterogeneity in rates across loci. Such a scenario involves a vast parameter space, which complicates its implementation in inferential settings. However, any structure in the data can be extracted as the covariation in rates in subsets of loci and subsets of branches across continuous gradients, creating “axes” of variation (Fig. 1e). Testing hypotheses of branch contributions along such rate axes offers a high-level view of variation in rates across the genome and across a phylogeny (Fig. 2). ClockstaRX implements such tests in R and includes step-by-step tutorials through github.com/duchene/ClockstaRX (supplementary fig. S1, Supplementary Material online). The code and results of the simulations study are available in github.com/duchene/crxTests.

**Fig. 2.**
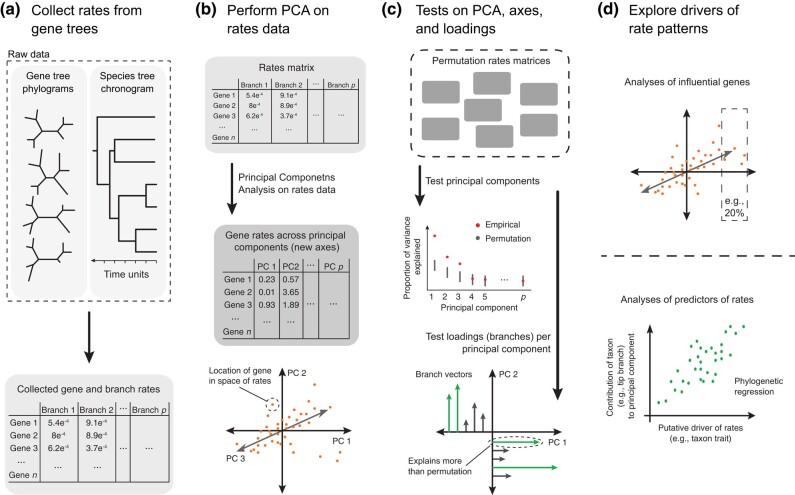
Framework of analysis of rates data using ClockstaRX. (a) Raw data are collected from gene trees and the species tree into a rates matrix that can include missing data due to incongruence between gene trees and the species tree. (b) The rates data are then analyzed using PCA, identifying the axes of genes with correlated rates across branches. (c) This PCA, each axis, and its loadings can all be assessed for explaining more information than expected under permutation. (d) These insights can be used for various further analyses. Genes with distinct signals along subsets of branches can be modeled differently for molecular dating and examined for their function. Similarly, lineages that stand out along axes can be examined for any important correlates of traits (traits or environment).

## Collection of Rates Data

The basic input for ClockstaRX is an inferred species tree and a set of gene trees with branch lengths representing expected substitutions per site. The species tree can be a rooted time-tree, which allows the gene-tree branch lengths to be converted into branch rates, or can be unrooted so that raw gene-tree branch lengths are used instead of data on rates. Gene trees are the phylogenetic inferences from individual loci, separated by putative recombination breakpoints. Gene trees are assumed to be independent unrooted phylograms, such that their branch lengths scale with the amount of evolution (e.g. expected substitutions per site). Therefore, rates across genes (or genomic loci) and across branches are assumed to be inferred independently.

ClockstaRX follows concordance-based collection of molecular rates (Walker et al. 2022), extracting the rate from each phylogenetic branch in each gene tree if it does not conflict with the species-tree topology (function collect.clocks). This approach is an effective method that bypasses sources of bias associated with analyses of rates (Walker et al. 2022), such as from missing taxa in some gene trees, from node-density effects (Hugall and Lee 2007), and from gene-tree discordance due to incomplete lineage sorting or gene-tree estimation error (Mendes and Hahn 2016).

## Modeling the Euclidean Space of Rates

ClockstaRX implements two approaches for describing the high dimensionality of rates across branches and loci. The first approach follows its predecessor, ClockstaR, in using multidimensional scaling (MDS) to map gene trees into two dimensions (Duchêne et al. 2014), followed by grouping loci into *k* clusters of rates (function group.clocks; supplementary fig. S4, Supplementary Material online; Kaufman and Rousseeuw 1990). The second and preferred method in ClockstaRX is to model the main axes of variation in rates using principal components analysis (PCA). The *n* × *p* matrix used in PCA consists of the *n* loci across rows and the *p* branches of the species tree as columns (function clock.space). In addition to the method of extracting branch lengths from genes, the emphasis in ClockstaRX on PCA and associated tests is the primary distinction from its predecessor. This approach minimizes the distortion of the space of rates arising from MDS and allows for formal tests on PCA axes and branch loadings.

Under the proposed framework, each principal component (PC) can incorporate the variance in rates across all branches simultaneously. The first PC is a model of the maximal correlation across branches. The loadings on a PC measure the correlation of each branch with the axis. Similarly, the magnitudes of loci in a PC indicate the relative rates of these loci on the branches with high loadings. In practice, if a single PC drives most of the variation in rates, molecular dating can be performed using a simple model that allows each locus to have a distinct relative rate. Alternatively, if multiple PCs explain large amounts of the variance (see tests below), then it is advisable to identify the subsets of loci that are disproportionately influencing rates in subsets of branches. The evolution of these loci can be modeled independently (e.g. by partitioning them to “unlink” their rates), or the loci can be excluded from analyses if their rate patterns are too complex to model.

## Evaluating Pacemaker Models

Describing molecular clocks using PCA allows additional tests of molecular clock hypotheses. ClockstaRX implements three types of tests (function clock.space), starting with a test of the degenerate multiple pacemaker model of genome evolution (Snir et al. 2012), where the null hypothesis is that of fully independent rates across loci and branches (results found in pca.clock.space$pPhi and pca.clock.space$pPsi).

The test statistics implemented, *ϕ* and *ψ*, use the magnitudes of PCA eigenvalues to estimate the overall degree of correlation between variables (Vieira 2012; Björklund 2019). The degree of covariation in the empirical data is then compared with that under permutation of samples (in this case the rate at each locus) within each variable (in this case the branches). A significant result from this test indicates that there is a predictable component of rate variation across branches and loci, rejecting the degenerate multiple pacemaker model.

A second test identifies the number of pacemakers required to describe the data (results found in pca.clock.space$pPCs), and specifically the number of PCs that significantly describe variation in the data. A PC that describes a greater proportion of the variance than the permuted sample can be considered as a variable that significantly describes evolutionary rate variation, and therefore a “pacemaker”.

A third test identifies local pacemakers by evaluating whether each branch is significantly contributing to the signal at each PC (pca.clock.space$pIL). This is done by testing whether the variable loadings at each PC are greater than those under permutation. Therefore, this test evaluates whether specific branches have a significantly greater influence on each PC than expected under permutation, and are thus driving variation in evolutionary rates. In molecular dating, these tests can be used in combination for defining the best scheme for modeling rate variation. For instance, if the tests of *ϕ* and *ψ* are significant and only a single PC significantly explains variation, then it is advisable to use a single clock while allowing each locus to have a distinct relative rate (following the universal pacemaker model).

Branches or loci that stand out for their contribution to variance can have negative impacts on molecular dating, in particular when multiple taxa and loci stand out across different PCs. This can be investigated by performing additional dating analyses that exclude any data that complicate rate estimation. The data that might be excluded and explored further might include the 20% of loci at the extremes of PCs or highly influential branches. In studies of the drivers of rates across branches, the loci at the extremes of significant PCs can be scrutinized for their functional roles or genomic location. Therefore, examining the Euclidean space with the aid of these pacemaker tests allows for better-informed study of phylogenetic dating and of the drivers of molecular evolution.

## Testing Correlates of Variation

ClockstaRX provides basic visualization of possible correlates of locus rates in Euclidean space. By default, the output includes the data across the first two PCs, colored by a variety of metrics associated with the gene trees used as input. These metrics include the pattern of clustering of loci, overall locus rate, and mean branch support per locus. Some metrics can have a strong association with evolutionary rate variation. In addition, the user can provide any number of other variables that might be associated with the distribution of gene trees in Euclidean space, such as whether they represent coding or noncoding loci, their chromosome, or whether they include specific taxa or internal branches of interest.

## Simulation Study

We used simulations to demonstrate the extreme scenarios where ClockstaRX can have reduced performance in identifying rate patterns in the data. We generated a species tree under a birth-death process (*N* = 50 tips; age = 50 time units; *λ* = 0.5; *μ* = 0.1) using *TreeSim*, which uses the number of tips as a stopping criterion and then scales the times in the resulting tree (Stadler 2011). Gene trees were then simulated as embedded in each species tree under the multispecies coalescent with a constant population size as implemented in *phybase* (Liu and Yu 2010). The factors that varied in simulations included: the degree of gene-tree discordance arising from incomplete lineage sorting (*θ* = 0.02, 0.5, or 1); the mean overall rate (0.01, 0.05, or 0.1 substitutions per site per time unit); the extent of among-lineage rate variation (mean standard deviation = 0.005, 0.05, and 0.5) under a white-noise molecular clock model (Lepage et al. 2007), as implemented in *NELSI* (Ho et al. 2015); number of loci sampled (100, 500, or 1000); the number of clock clusters (*k* = 1, 3, or 5); and a portion of branches (0%, 2%, or 10% of all branches) having rates accelerated by a factor of five. We performed ten simulations for each of the 729 scenarios. We then assessed the accuracy of ClockstaRX in identifying dimensional reducibility as tested by the *ϕ* and *ψ* tests, the number of clusters *k* in the data, and the accuracy in identification of branches with accelerated rates.

Our simulation study shows that the *ϕ* and *ψ* tests of the degenerate multiple pacemaker are influenced by among-lineage rate heterogeneity and by how loci are clustered by their patterns of rates among branches (supplementary table S1, Supplementary Material online). The software has the expected behavior of rejecting the degenerate multiple pacemaker model in data with intermediate amounts of among-lineage rate heterogeneity (supplementary fig. S2, Supplementary Material online). In cases where rates data collapse to the center of the Euclidean space, or become too spread out, the test will support a degenerate model where each locus has a fully independent signal.

**Fig. 3.**
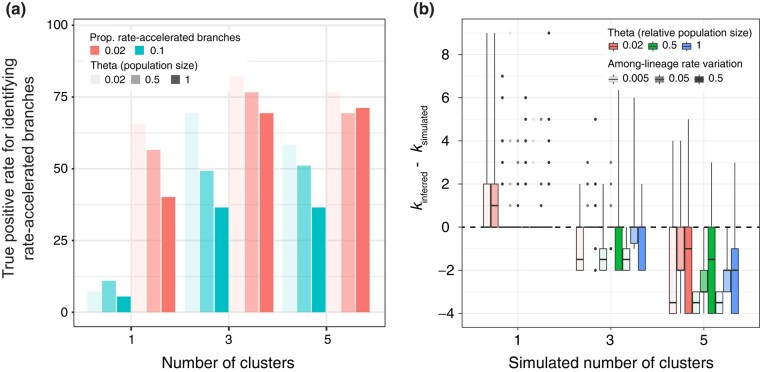
Primary results from simulations of molecular rates. (a) Identifying rate-accelerated lineages is easiest in broadly spread data (e.g. due to clustering), when only a few branches have accelerated rates, and when there is limited missing data (e.g. due to incomplete lineage sorting represented in high *θ*). (b) Correctly identifying the number of clusters is generally difficult, a result that supports the shift away from clustering in ClockstaRX and toward an assessment of rate axes using PCA. Large amounts of missing data are also detrimental for identifying clusters, while large amounts of additional among-lineage rate variation leads to greater variance in clustering inferences.

Branches that were simulated to have accelerated rates were identified most accurately when the rate acceleration was confined to a small portion of branches, and when minimal amounts of data were missing due to incomplete lineage sorting (Fig. 3a). As expected, branches that have experienced significant rate accelerations can be identified more easily if they stand out from other forms of structure, or noise, in the data.

Our simulations show that identifying multiple clusters of gene trees (*k*) in the Euclidean space of rates is difficult overall (Fig. 3), such that it is preferable to explore data by testing influential axes and branches rather than via clustering. Nonetheless, the gap statistic implemented in ClockstaRX for the selection of the number of clusters (also proposed earlier by Duchêne et al. 2018a; Duchêne et al. 2018b) had greater accuracy than other existing criteria (supplementary fig. S3, Supplementary Material online).

Given these results, we advise users to first examine their data for loci and taxa with potentially misleading signals (Vankan et al. 2022). One example is by using Felsenstein's likelihood-ratio test for testing large departures from clocklike evolution. Similarly, analyses might benefit from excluding unusually long branches, which can be done by assuming that branches follow some distribution (e.g. exponential) and excluding those that fall in the tail (e.g. *P* < 0.01; also see Mai and Mirarab 2018). Good practice also involves verifying that loci are sufficiently variable to allow rates to be inferred reliably (Dornburg et al. 2019; Duchêne et al. 2022). Loci might also be excluded if branch supports are below a chosen threshold (e.g. mean < 0.9), or if they fail model-adequacy tests from software such as PhyloMAd (Duchêne et al. 2018a; Duchêne et al. 2018b) or IQTREE2 (Bui et al. 2020).

In conclusion, ClockstaRX provides a user-friendly tool for achieving comprehensive descriptions of molecular rates in phylogenomic data, allowing insights into the drivers of evolutionary rates and aiding the construction of models for molecular dating. In addition to hypothesis testing and visualization of clock patterns, the results can naturally be interpreted in the context of biological trait and gene function data, allowing identification of the drivers of rate variation. By placing molecular clocks into a comparative analytical framework, ClockstaRX aims to expand our understanding of evolutionary rates and timescales across genes and lineages in the Tree of Life.

## Supplementary Material

evae064_Supplementary_Data

## Data Availability

The code for software and simulations presented in this article are available in github.com/duchene/ClockstaRX and github.com/duchene/crxTests, respectively. No new biological data were generated or analyzed in support of this research.
