# Supplementary material for: ClockstaRX: Testing Molecular Clock Hypotheses With Genomic Data
Source: Genome Biol Evol. 2024 Mar 25;16(4):evae064. doi: 10.1093/gbe/evae064 (PMC10999959; doi:10.1093/gbe/evae064)
Supplement: evae064_Supplementary_Data [file evae064_supplementary_data.docx]

# Supplementary material

# ClockstaRX: testing molecular clock hypotheses with genomic data

David A. Duchêne^1^*, Sebastián Duchêne^2^, Josefin Stiller^3^, Rasmus Heller^4^, and Simon Y. W. Ho^5^

^1^Center for Evolutionary Hologenomics, University of Copenhagen, 1352 Copenhagen, Denmark

^2^Department of Microbiology and Immunology, Peter Doherty Institute for Infection and Immunity, University of Melbourne, Melbourne, VIC 3010, Australia

^3^Villum Centre for Biodiversity Genomics, University of Copenhagen, 2100 Copenhagen, Denmark

^4^Section for Computational and RNA Biology, Department of Biology, University of Copenhagen, 2100 Copenhagen, Denmark

^5^School of Life and Environmental Sciences, University of Sydney, Sydney, NSW 2006, Australia

**Table S1.** *P*-values of regression analyses testing the association between each of the simulation variables (columns) and eight estimates from ClockstaRX (rows).

|  | Theta (relative population size) | Number of loci | Among-lineage rate variation | Proportion of lineages with accelerated rate | Number of clock clusters | Mean overall rate | Clustering type (overall rate or clock) |
| --- | --- | --- | --- | --- | --- | --- | --- |
| *k* | <0.0001 | 0.0312 | 0.0105 | 0.1153 | 0.1718 | 0.1131 | 0.0027 |
| *k*_inferred_ - *k*_simulated_ | <0.0001 | 0.0946 | 0.0472 | 0.2217 | <0.0001 | 0.2189 | 0.0201 |
| *k* (weighted clock data) | <0.0001 | 0.0024 | 0.0006 | 0.0420 | <0.0001 | 0.9807 | 0.1987 |
| *k*_inferred_ - *k*_simulated_ (weighted clock data) | <0.0001 | 0.0127 | 0.0048 | 0.0955 | <0.0001 | 0.9842 | 0.2918 |
| Success in identifying influential lineages  (TRUE / FALSE) | 0.0005 | <0.1110 | 0.1722 | <0.0001 | <0.0001 | 0.6442 | NA |
| Influential lineages false-positive rate | 0.0820 | <0.0001 | 0.0053 | 0.0018 | <0.0001 | 0.7448 | 0.0070 |
| *φ* statistic | 0.1717 | 0.0691 | <0.0001 | 0.0845 | 0.0658 | 0.5646 | 0.0191 |
| *ψ* statistic | 0.1798 | 0.0640 | <0.0001 | 0.0830 | 0.0669 | 0.5632 | 0.0200 |


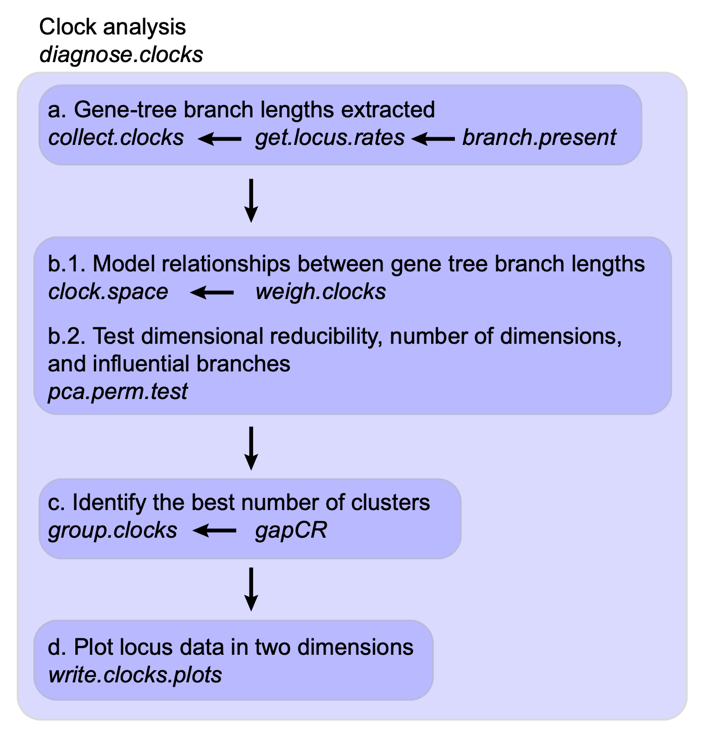


**Figure S1.** Workflow for ClockstaRX analyses. Main function names are shown in italics.

**
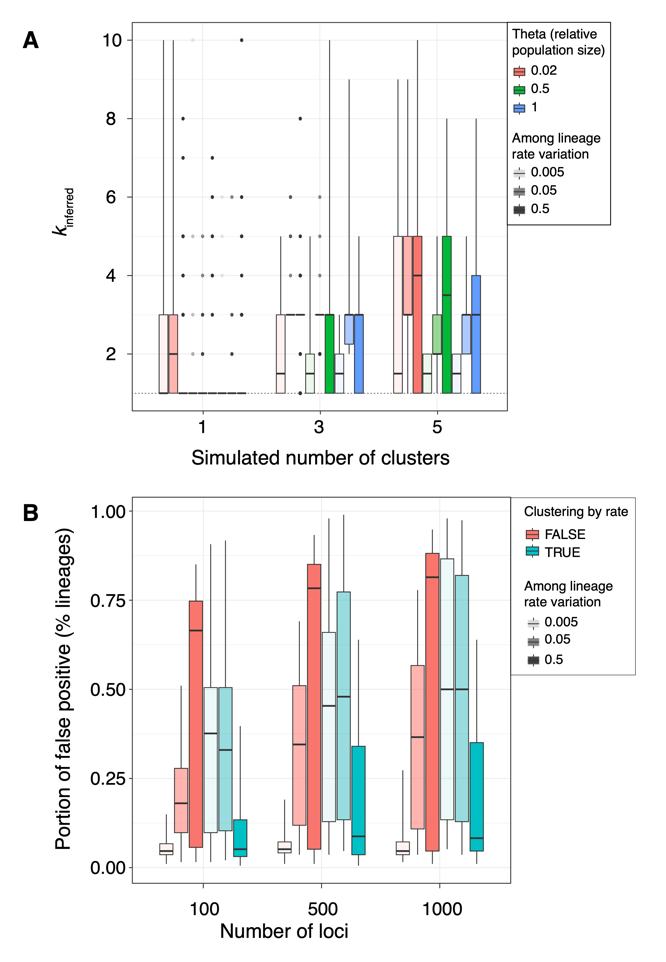
**

**Figure S2.** Inferences from ClockstaRX from data simulated under a range of scenarios of evolutionary rates across loci and lineages. Inferences of interest include (a) number of clusters inferred, and (b) portion of lineages that were not synthetically accelerated yet were found to significantly contribute to variance (false positives).


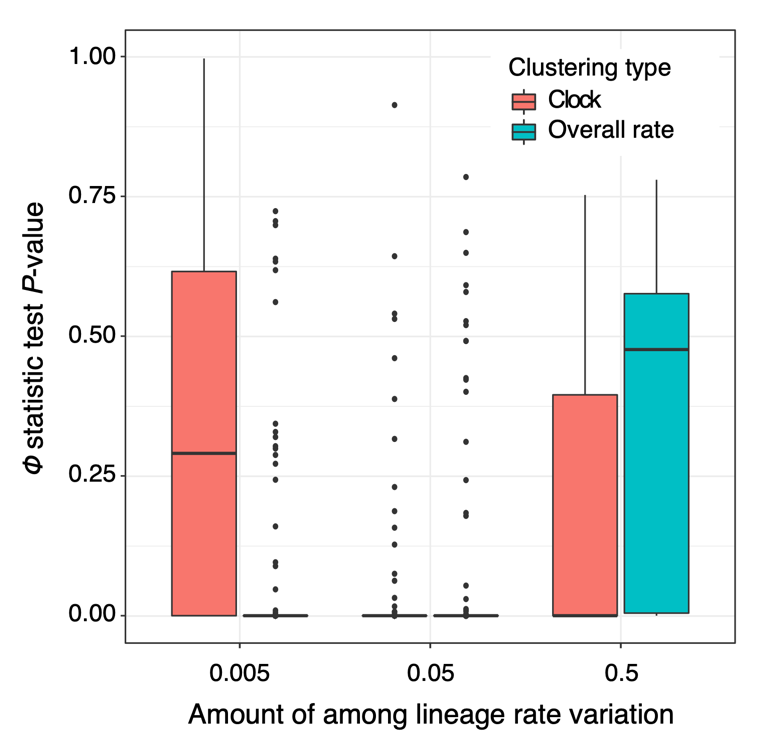


**Figure S3.** Results of the *Φ*-test of dimensional reducibility across simulations, focusing on the two simulation factors that best explained the test results. Intermediate amounts of among-lineage rate variation show the best performance, frequently rejecting the null hypothesis of a lack of correlation in the data (*P*-value < 0.01). Extremely high or low values of among lineage rate variation show generally poor performance. One exception is that of scenarios of clustering by patterns of among-lineage rate variation and low overall variance in rates, which leads to frequent rejection of the null.


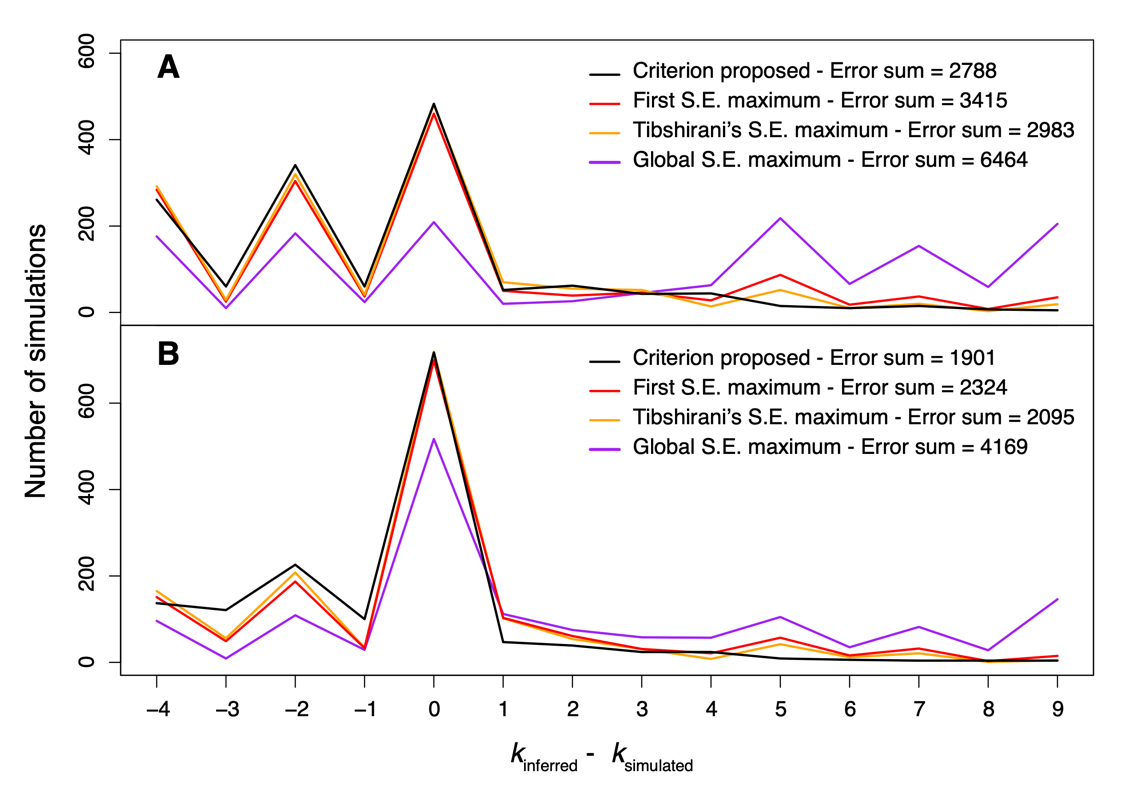


**Figure S4.** Comparison of metrics for selecting *k*, showing the number of simulations (y-axis) in which each difference between inferred and simulated *k* was found (x-axis). The error scores shown in the legend are the sum of the absolute x-axis values for each metric, such that increasing values are equivalent to a greater departure on average from the simulated *k*.
